# Spatial Entanglement of Fermions in One-Dimensional Quantum Dots

**DOI:** 10.3390/e23070868

**Published:** 2021-07-07

**Authors:** Ivan P. Christov

**Affiliations:** 1 Physics Department, Sofia University, 1164 Sofia, Bulgaria; ivan.christov@phys.uni-sofia.bg; 2 Institute of Electronics, Bulgarian Academy of Sciences, 1784 Sofia, Bulgaria

**Keywords:** quantum correlations, quantum entanglement, quantum Monte Carlo method

## Abstract

The time-dependent quantum Monte Carlo method for fermions is introduced and applied in the calculation of the entanglement of electrons in one-dimensional quantum dots with several spin-polarized and spin-compensated electron configurations. The rich statistics of wave functions provided by this method allow one to build reduced density matrices for each electron, and to quantify the spatial entanglement using measures such as quantum entropy by treating the electrons as identical or distinguishable particles. Our results indicate that the spatial entanglement in parallel-spin configurations is rather small, and is determined mostly by the spatial quantum nonlocality introduced by the ground state. By contrast, in the spin-compensated case, the outermost opposite-spin electrons interact like bosons, which prevails their entanglement, while the inner-shell electrons remain largely at their Hartree–Fock geometry. Our findings are in close correspondence with the numerically exact results, wherever such comparison is possible.

## 1. Introduction

During the past few decades there has been an increasing interest in developing new models and computational tools to address the fundamental and practical challenges related to quantum correlations and entanglement, in connection with their potential application in newly emerging quantum technologies [1]. The properties of composite systems of quantum particles are expected to play an important role in information processing, as well as in devices for manipulating systems of atoms and molecules. While various algebraic operator methods have been used to characterize entanglement in spin systems [2,3,4], an efficient approach to assess the spatial entanglement in many-body quantum systems together with its evolution over time is still lacking. It is well known that the correlated non-relativistic particle motion described by the time-dependent Schrödinger equation (SE) is tractable for only a limited number of cases. While solvable numerically for few particles in 1D and 2D, the direct numerical solution of the SE scales exponentially with system size, and is therefore beyond the capabilities of today’s computers. That exponential time scaling is usually attributed to the nonlocal quantum effects that result from the dependence of the wave function $\Psi (r_{1},\ldots ,r_{N},t)$ on the coordinates of all interacting particles. The standard approaches to ameliorate the workload are to reduce the many-body SE to a set of coupled single-body equations, where the most prominent are the mean-field approaches: the Hartree–Fock (HF) method [5] and density-functional theory (DFT) [6]. That reduction, however, occurs at the price of neglecting the detailed fluctuating forces between the electrons and replacing them with averages, thus totally ignoring the dynamic quantum correlations in the HF method, while DFT reduces the many-body problem to a single-body problem of non-interacting electrons moving in an effective exchange-correlation potential, which is generally unknown and suffers self-interaction issues. More accurate but more computationally expensive are the multi-configuration time-dependent Hartree–Fock method [7], and the full configuration interaction method [8]. Another approach that has gained much attention lately is the density matrix renormalization group method [9], which allows one to treat correlated 1D many-body problems with good accuracy; however, its application to higher dimensions and arbitrary potentials has been challenging thus far.

A different class of methods to tackle the quantum many-body problem includes the quantum Monte Carlo methods [10], which allow one to accurately calculate the electronic structures of atoms, molecules, nanostructures, and condensed-matter systems at a fully correlated level. For example, the diffusion quantum Monte Carlo (DMC) method uses random particles (walkers) whose evolution towards the ground state of the system involves a combination of diffusion and branching—which, however, prevent its use for real-time-dependent processes where causality is of primary importance. Moreover, the artificial nature of the many-body wave function in configuration space used in the DMC method prevents the calculation of some important quantities other than the energy. The recently introduced time-dependent quantum Monte Carlo (TDQMC) method [11,12,13] employs concurrent ensembles of walkers and wave functions for each electron, where each wave function is associated with a separate walker (particle–wave dichotomy), and these evolve in physical spacetime where no initial guess for the many-body wave function is needed. Recently, we have applied the TDQMC method to analyze the ground state preparation for simple bosonic systems with several particles in one and two dimensions, with a good tradeoff between scaling and accuracy [14]. In this work we apply the TDQMC method to several interacting fermions in one-dimensional quantum dots, where the obtained reduced density matrices allow us to quantify the entanglement between the electrons considered to be identical or distinguishable particles. We consider some proof-of-principle aspects of the TDQMC method for fermions, rather than practical matters concerning quantum dots. Entanglement in two-electron quantum dots, atoms, and molecules has been studied elsewhere [15,16,17,18].

## 2. Methods

The TDQMC method transforms the standard Hartree–Fock (HF) equations into a set of coupled stochastic equations capable of describing the correlated particle motion. That transformation is based on the physical assumption that the modulus square of the single-body wave function in coordinate (physical) space may be thought of as an envelope (or kernel density estimation) of the distribution of a finite number of particles (walkers). In this way, for each electron in an atom a large set of single-body wave functions that reside in physical spacetime is created, where each wave function responds to the multicore potential due to both the nucleus and the walkers of the rest of the electrons. The crucial point in this picture is that it allows for each walker for a given electron to interact with the walkers of any other electron through weighted Coulomb potential, thus naturally incorporating the spatial quantum nonlocality. Then, from the evolution of the walker distributions one can evaluate quantum observables without resorting to the many-body wave function. Formally, we start from the HF equation for the i^th^ from a total of N electrons, within a single-determinant ansatz [19]:

(1) $$i\hbar \frac{\partial }{\partial t}\varphi_{i}(r_{i},t)=\left[-\frac{\hbar^{2}}{2m}\nabla_{i}^{2}+V_{en}(r_{i})+V_{ee}^{HF}(r_{i},t)\right]\varphi_{i}(r_{i},t)$$

where $V_{en}(r_{i})$ is the electron–nuclear potential, and the HF electron–electron potential reads:

(2) $$V_{ee}^{HF}\left(r_{i},t\right)=V_{ee}^{H}\left(r_{i},t\right)+V_{ee}^{X}\left(r_{i},t\right)$$

where:

(3) $$V_{ee}^{H}\left(r_{i},t\right)=\sum_{j\neq i}^{N}\int dr_{j}V_{ee}(r_{i}-r_{j}){\left|\varphi_{j}(r_{j},t)\right|}^{2}$$

is the Hartree potential, and $V_{ee}^{X}\left(r_{i},t\right)$ is the exchange potential:

(4) $$V_{ee}^{X}\left(r_{i},t\right)=-\sum_{j\neq i}^{N}\delta_{s_{i},s_{j}}\int dr_{j}V_{ee}(r_{i}-r_{j})\varphi_{i}(r_{j},t)\varphi_{j}^{*}(r_{j},t)\varphi_{j}(r_{i},t)/\varphi_{i}(r_{i},t)$$

where $\varphi_{i}(r,t)$ satisfy the orthonormality property $\int \varphi_{i}(r,t)\varphi_{j}^{*}(r,t)dr=\delta_{i,j}$, and the indices $s_{i},s_{j}$ denote the spins of the corresponding electrons. The inequality j≠i in the sums of Equations (3) and (4) stresses the fact that even though the self-interaction between the electrons is naturally canceled in the HF approximation, it is also not present in the Hartree approximation, where there is no exchange potential [19]. It is known that the wave functions $\varphi_{i}(r,t)$ of Equation (1) variationally minimize the system energy:

(5) $$E^{HF}=\sum_{i=1}^{N}\left[-\frac{\hbar^{2}}{2m}\int \varphi_{i}^{*}\left(r_{i},\tau \right)\nabla_{i}^{2}\varphi_{i}\left(r_{i},\tau \right)dr_{i}+\int V_{en}(r_{i}){\left|\varphi_{i}\left(r_{i},\tau \right)\right|}^{2}dr_{i}\right]+E_{ee}^{H}+E_{ee}^{X}$$

where the Hartree and exchange energies read:

(6) $$E_{ee}^{H}=0.5\sum_{i\neq j}^{N}\iint V_{ee}(r_{i}-r_{j}){\left|\varphi_{i}\left(r_{i},\tau \right)\right|}^{2}{\left|\varphi_{j}\left(r_{j},\tau \right)\right|}^{2}dr_{i}dr_{j}$$

and

(7) $$E_{ee}^{X}=-0.5\sum_{i\neq j}^{N}\delta_{s_{i},s_{j}}\iint V_{ee}(r_{i}-r_{j})\varphi_{i}(r_{j},\tau )\varphi_{j}^{*}(r_{j},\tau )\varphi_{j}(r_{i},\tau )\varphi_{i}^{*}(r_{i},\tau )dr_{i}dr_{j}$$

respectively.

It is known that the Hartree–Fock approximation does not account for the dynamic electron–electron correlations beyond those due to the exchange interaction. In order to correct for this in the TDQMC methodology, we replace the HF wave function for each electron $\varphi_{i}(r,t)$ with a family of slightly different wave functions $\varphi_{i}(r,t)\to \varphi_{i}^{k}(r,t)$; k = 1,…,M [11,12,13], which allows us to further lower the system energy below the HF level. This is accomplished by applying a stochastic windowing to the distribution ${\left|\varphi_{j}(r_{j},t)\right|}^{2}$ in the Hartree potential $V_{ee}^{H}\left(r_{i},t\right)$ of Equation (3), by using a “window” function $Κ\left[r_{j},r_{j}^{k}(t),\sigma_{j,i}\right]$ centered at a certain walker’s trajectory $r_{j}^{k}(t)$, which samples the distribution given by ${\left|\varphi_{j}(r_{j},t)\right|}^{2}$. The parameters $\sigma_{j,i}$ determine the widths of those “windows”, such that the product ${\left|\varphi_{j}(r_{j},t)\right|}^{2}Κ\left[r_{j},r_{j}^{k}(t),\sigma_{j,i}\right]$ is different for each separate trajectory $r_{j}^{k}(t)$. In this way, for each electron, Equations (1)–(4) are transformed into a set of M Hartree–Fock-like equations for the different replicas $\varphi_{i}^{k}(r_{i},t)$ of the initial HF wave function $\varphi_{i}(r_{i},t)$, each one attached to a separate trajectory $r_{j}^{k}(t)$ (particle–wave dichotomy [13]):

(8) $$\begin{array}{l} i\hbar \frac{\partial }{\partial t}\varphi_{i}^{k}(r_{i},t)=\left[-\frac{\hbar^{2}}{2m}\nabla_{i}^{2}+V_{en}(r_{i})+V_{eff}^{k}\left(r_{i},t\right)\right.\\ \left.-\sum_{j\neq i}^{N}\delta_{s_{i},s_{j}}\int dr_{j}V_{ee}(r_{i}-r_{j})\varphi_{i}^{k}(r_{j},t)\varphi_{j}^{k*}(r_{j},t)\varphi_{j}^{k}(r_{i},t)/\varphi_{i}^{k}(r_{i},t)\right]\varphi_{i}^{k}(r_{i},t) \end{array}$$

where $\int \varphi_{i}^{k}(r,t)\varphi_{j}^{k*}(r,t)dr=\delta_{i,j}$; i = 1,…,N, k = 1,…,M, and where:

(9) $$V_{eff}^{k}\left(r_{i},t\right)=\sum_{j\neq i}^{N}\frac{1}{Z_{j,i}^{k}}\sum_{l}^{M}V_{ee}\left[r_{i},r_{j}^{l}(t)\right]Κ\left[r_{j}^{l}(t),r_{j}^{k}(t),\sigma_{j,i}\right]$$

is the effective electron–electron interaction potential represented as a Monte Carlo (MC) convolution that incorporates the spatial quantum nonlocality by allowing each walker for a given electron to interact with a group of walkers of any other electron. In fact, it can be seen from Equation (9) that the effective potential “seen” by the *k*th wave function for the *i*th electron involves interactions with a number of walkers that belong to the *j*th electron and lie within the nonlocal length $\sigma_{j,i}$ around $r_{j}(t)$ [12,13,14]. For the Gaussian kernel we have:

(10) $$Κ\left[r_{j},r_{j}^{k}(t),\sigma_{j,i}\right]=\exp\left(-\frac{{\left|r_{j}-r_{j}^{k}(t)\right|}^{2}}{2\sigma_{j,i}^{2}}\right)$$

which determines the weighting factor in Equation (9) to be:

(11) $$Z_{j,i}^{k}=\sum_{l=1}^{M}Κ\left[r_{j}^{l}(t),r_{j}^{k}(t),\sigma_{j,i}\right]$$

As seen from Equations (10) and (11), the limit $\sigma_{j,i}\to \infty$ where $Κ\left[r_{j},r_{j}^{k}(t),\sigma_{j,i}\right]\to 1$ recovers the Hartree–Fock approximation—as opposed to the local interaction, where $\sigma_{j,i}\to 0$ and $Κ\left[r_{j},r_{j}^{k}(t),\sigma_{j,i}\right]\to \delta \left(r_{j}-r_{j}^{k}(t)\right)$. It is clear, therefore, that $\sigma_{j,i}$ may serve as variational parameters to minimize the system energy between these two limiting cases.

The connection between the trajectories $r_{i}^{k}(t)$ and the wave functions $\varphi_{i}^{k}(r_{i},t)$ is given by the walker’s velocities (de Broglie–Bohm equation, e.g., in [20]):

(12) vikt==ℏmIm∇iφik(ri,t)φik(ri,t)ri=rik(t)

for real-time propagation, and:

(13) $$dr_{i}^{k}\left(\tau \right)=v^{D}{}_{i}^{k}d\tau +\eta_{i}\left(\tau \right)\sqrt{\frac{\hbar }{m}d\tau }$$

for imaginary-time propagation, where the drift velocity reads:

(14) $$v^{D}{}_{i}^{k}\left(\tau \right)=\frac{\hbar }{m}\;{\left[\frac{\nabla_{i}\varphi_{i}^{k}(r_{i},\tau )}{\varphi_{i}^{k}(r_{i},\tau )}\right]}_{r_{i}=r_{i}^{k}(\tau )}$$

and $\eta \left(\tau \right)$ is a Markovian stochastic process (see also the appendix in [21]). The striking similarity between the drift velocity of Equation (14) and the de Broglie–Bohm Equation (12) comes from the fact that both equations describe drift-diffusion processes in imaginary and in real time, respectively. It is seen that although the individual wave functions guide the corresponding walkers through Equation (12), the TDQMC method solves coupled single-body Hartree-Fock-like equations (e.g., Equation (8)) instead of using quantum potentials as in Bohmian mechanics [20]. 

Following the particle–wave dichotomy described above, the system energy can be calculated conveniently using both particle trajectories and wave functions:

(15) $$E=\frac{1}{M}{\sum_{k=1}^{M}\left[\sum_{i=1}^{N}\left[-\frac{\hbar^{2}}{2m}\frac{\nabla_{i}^{2}\varphi_{i}^{k}(r_{i}^{k})}{\varphi_{i}^{k}(r_{i}^{k})}+V_{en}(r_{i}^{k})\right]+\sum_{i>j}^{N}V_{ee}(r_{i}^{k}-r_{j}^{k})\right]}_{\begin{array}{l} r_{i}^{k}=r_{i}^{k}(\tau )\\ r_{j}^{k}=r_{j}^{k}(\tau ) \end{array}}\;-\frac{0.5}{M}\sum_{k=1}^{M}\sum_{i\neq j}^{N}\delta_{s_{i},s_{j}}\int \int dr_{i}dr_{j}V_{ee}(r_{i}-r_{j})\varphi_{i}^{k}(r_{j},t)\varphi_{j}^{k*}(r_{j},t)\varphi_{j}^{k}(r_{i},t)\varphi_{i}^{k*}(r_{i},t).$$

During the preparation of the ground state of the quantum system, the initial Monte Carlo ensembles of walkers and wave functions propagate in imaginary time (τ) toward a steady state, in accordance with Equations (8)–(14), for different values of the nonlocality parameters $\sigma_{j,i}$, until the energy of Equation (15) displays a minimum. Another alternative to account for the exchange effects is to apply a short-range screening to the interaction potential in order to modify the repulsion between the same-spin electrons [22].

Considering the ensemble of waves $\varphi_{i}^{k}(r_{i},t)$ delivered by the TDQMC as random variables, one can build a reduced density matrix for the *i*th electron, which may serve as the variance–covariance matrix in the Hilbert space that carries important statistical information [13,23]:

(16) $$\rho_{i}\left(r_{i},{r^{\prime }}_{i},t\right)=\frac{1}{M}\sum_{k=1}^{M}\varphi_{i}^{k}{}^{*}(r_{i},t)\varphi_{i}^{k}({r^{\prime }}_{i},t)$$

For example, the density matrix of Equation (16) allows one to easily calculate the spatial entanglement of a given electron state, which may serve also as a good measure for the overall accuracy of the calculation (e.g., [24]). Without entering the ongoing debate on entanglement witnesses, for opposite-spin electrons—where there are no exchange terms in HF and TDQMC equations—we employ the linear quantum entropy for distinguishable (non-identical) particles as a conventional measure for the spatial entanglement [25]:

(17) $$S_{L↑↓}^{i}\left(t\right)=1-Tr\left(\rho_{i}^{2}\right)=1-\int \rho_{i}^{2}\left(r_{i},r_{i},t\right)dr_{i}$$

while for N same-spin electrons the component of the entanglement that reflects the trivial minimum correlation due to the anti-symmetrization of the wave function can be eliminated [26,27,28], yielding:

(18) $$S_{L}^{i}{}_{↑↑}\left(t\right)=1-NTr\left(\rho_{i}^{2}\right)=1-N\int \rho_{i}^{2}\left(r_{i},r_{i},t\right)dr_{i}$$

This definition ensures that for the wave functions used in HF approximation (or in general for any Slater rank 1 many-body state [29,30]) the linear entropy should vanish.

For indistinguishable (identical) particles, the spatial part of the 2N-body wave function can be represented in the simplest case as a product of normalized spin-up and spin-down Slater determinants [10]:

(19) $$\Psi_{i}(r_{1},r_{2},\ldots ,r_{2N},t)=D_{i}^{↑}(r_{1},r_{2},\ldots ,r_{N},t)D_{i}^{↓}(r_{N+1},r_{N+2},\ldots ,r_{2N},t)$$

Thus, the entanglement between—for example—spin-up only electrons can be estimated using the reduced density matrix, averaged over the configurations provided by the TDQMC algorithm:

(20) $$\rho_{i}^{↑}\left(r,r^{\prime },t\right)=\frac{1}{M}\sum_{k}^{M}\int D_{i}^{k↑}(r,r_{2},\ldots ,r_{N},t)D_{i}^{k↑}{}^{*}(r^{\prime },r_{2},\ldots ,r_{N},t)dr_{2}\ldots dr_{N}$$

where $D_{i}^{k↑}$ are spin-up Slater determinants composed by the individual wave functions.

## 3. Results

As an example here we calculate the ground state of a quantum dot with parabolic core potential $V_{en}(r_{i})=\omega^{2}r_{i}^{2}/2$ and with soft-core electron–electron Coulomb repulsion [31]:

(21) $$V_{ee}\left[r_{i},r_{j}\right]=\frac{e^{2}}{\sqrt{r^{2}+a^{2}}}$$

where $r\equiv \left|r_{i}-r_{j}\right|$. 

Within the formalism of Section 2 the degree of spatial correlation and, hence, the spatial entanglement, is controlled in TDQMC by the quantum nonlocal length $\sigma_{j,i}$ where, for bound electrons, higher $\sigma_{j,i}$ lead to lower correlation (entanglement) between the *i*th and the *j*th electron, and vice versa. Since the spatial extent of the electron cloud for the *j*th electron is determined by the standard deviation $s_{j}$ of the corresponding MC ensemble, the nonlocal length $\sigma_{j,i}$ is expected to be close to $s_{j}$:

(22) $$\sigma_{j,i}=\alpha_{j,i}.s_{j};\;j,i=1,\ldots ,N,$$

where $\alpha_{j,i}$ may now serve as the variational parameters to minimizing the energy.

**Figure 1:**
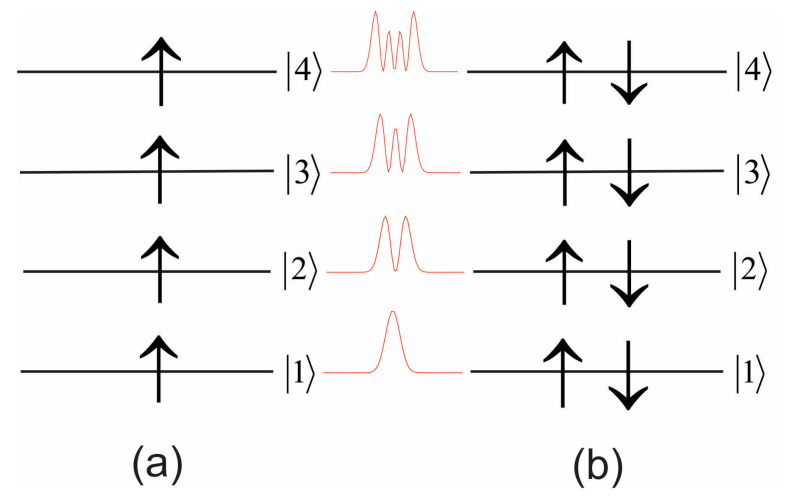
Energy level diagrams for spin-polarized (**a**) and spin-compensated (**b**) electrons in 1D quantum dots. The moduli square of the corresponding spatial orbitals are drawn with red.

Since for parallel-spin electrons the eigenstates are orthogonal to one another, their overlap is small and, hence, the dynamic correlation between such states is expected to be smaller compared to the correlation between opposite-spin electrons. Here, we consider 1D quantum dots in two basic configurations: one is a spin-polarized configuration where each energy level is filled with just one same-spin electron, as seen in Figure 1a, and the other is a spin-compensated configuration where each level is occupied by two opposite-spin electrons (Figure 1b).

**Figure 2:**
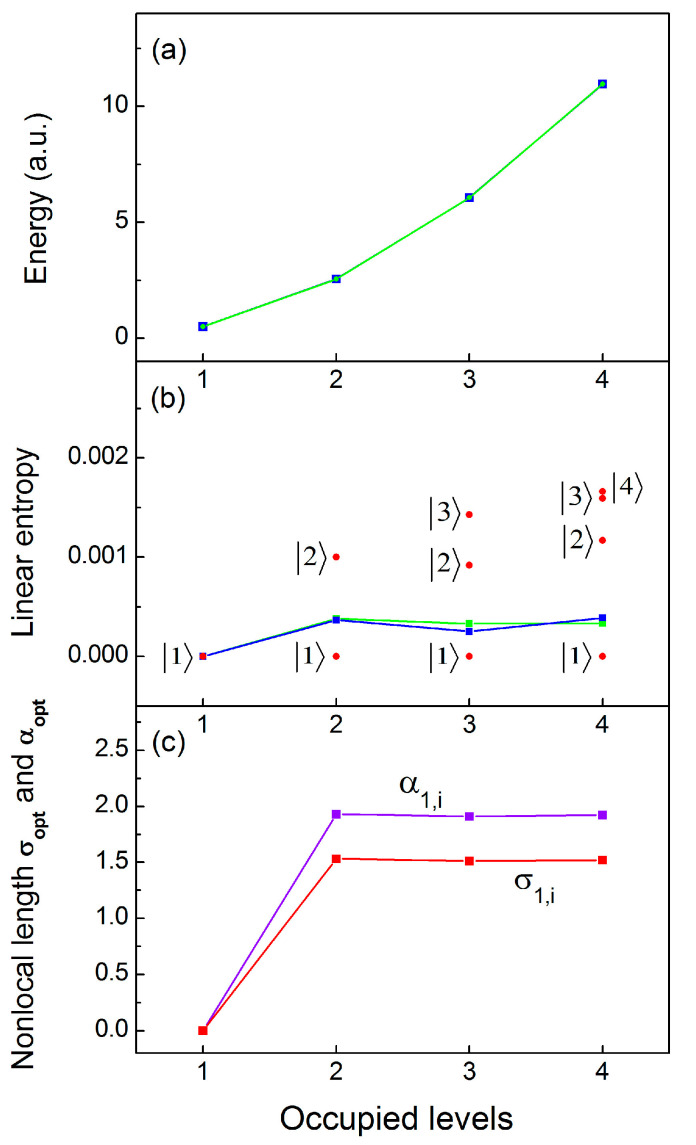
Energy (**a**), linear entropy (**b**), and nonlocality parameters $\sigma_{1,i}$ ($\alpha_{1,i}$) (**c**) for the ground state $|1\rangle$, for electron configurations with up to four parallel-spin electrons (Figure 1a). Blue lines: TDQMC results; green lines: numerically exact results; red dots in (**b**): linear entropy for distinguishable electrons.

We start with the ground state and the three excited states of a total of four same-spin electron configurations (Figure 1a), where due to the orthogonality of the spatial wave functions the dynamic correlations between the same-spin electrons are expected to be rather small. The system of coupled non-linear TDQMC equations (Equation (8)) is solved iteratively using both spilt-step Fourier and Crank–Nicolson numerical schemes, which give close results. The final states obtained are statistical variants of the corresponding Hartree–Fock wave functions for the different energy levels, with certain distortions due to the different effective interaction potentials $V_{eff}^{k}\left(r_{i},t\right)$ in Equation (8). Starting from preliminary calculated Hartree–Fock wave functions, after 200 steps of imaginary-time propagation of Equations (8)–(11) and Equations (13) and (14), for ω=1, and for different values of the variational parameter $\alpha_{j,i}$ of Equation (22), we find the energy minima for one, two, three, and four occupied levels in succession. Figure 2a shows these energies (green line), which are in a very good correspondence with the numerically exact energies (blue line) obtained from the direct numerical solution of the Schrödinger equation for up to four electrons in one spatial dimension. Our calculations reveal that accuracy of three significant digits for the energy can be attained by varying $\alpha_{1,i}$, while $\alpha_{2,i}$, $\alpha_{3,i}$, and $\alpha_{4,i}$ are set to infinity, which practically keeps the ground state (level 1) at its Hartree–Fock geometry. The optimal values of $\alpha_{1,i}$ for the ground level $|1\rangle$ are shown in Figure 2c for two, three, and four electrons, also showing that both $\alpha_{1,i}$ and the nonlocal length $\sigma_{1,i}$ are almost independent of the number of electrons—except for one electron at the ground state where there is no e–e interaction—and $\alpha_{1,i}$ is set to zero. The degree of entanglement for the four configurations of Figure 1a is quantified by the linear quantum entropy of Equation (18), as shown in Figure 2b. There are two distinct cases: In the first, the electrons are considered identical (blue line), to be compared with the result from the exact numerical solution of the Schrödinger equation (green line). It can be seen that the linear entropy in this case remains almost constant, in close agreement with the exact numerical result. The second case, plotted with red dots in Figure 2b, depicts the linear entropy for the different electrons considered as distinguishable particles with the density matrix of Equation (17). It can be seen that the linear entropy for the distinguishable electrons increases due to the screening effect of the inner electrons, which causes larger fluctuations in the shape of the outer wave functions.

**Figure 3:**
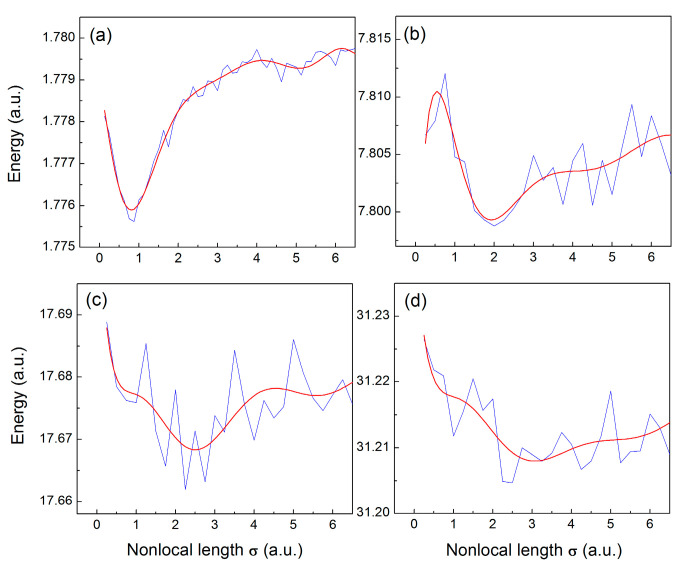
Energy of N-electron 1D quantum dots as a function of the nonlocal length $\sigma_{N,N}$, for N = 2 (**a**), N = 4 (**b**), N = 6 (**c**), and N = 8 (**d**).

For the filled-shell configuration of Figure 1b, each level contains two opposite-spin electrons, whose wave functions overlap in space almost completely, and therefore these electrons interact more like bosons [14]. It is therefore reasonable to calculate the only spatial entanglement that is due to same-shell states that is expected to significantly exceed the entanglement of the same-spin electrons at different levels. For the configuration of Figure 1b we have found that the major source of entanglement is the Coulomb repulsion between the two outermost electrons. For the ground state (level 1), where only two opposite-spin electrons are present in the vicinity of the core, the system energy exhibits a well-defined minimum as a function of the nonlocal length $\sigma_{1,1}^{↑}=\sigma_{1,1}^{↓}$, as seen in Figure 3a. When adding electrons at the higher levels, the corresponding wave functions acquire zeros, which is a source of larger fluctuations of the energy, as seen in Figure 3b–d, where the red curves represent the polynomial least squares fit for better visualization of the energy minimum.

**Figure 4:**
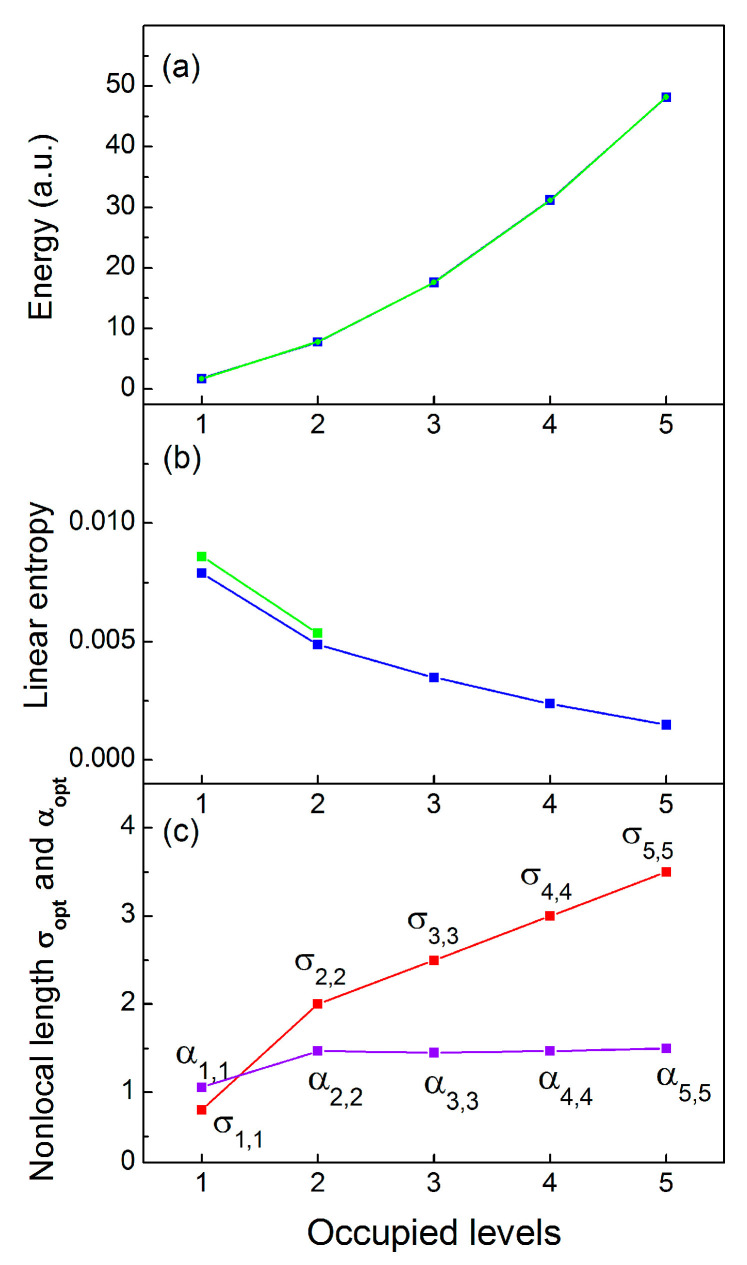
Energy (**a**), linear quantum entropy (**b**), and nonlocality parameters $\sigma_{1,i}$ ($\alpha_{1,i}$) (**c**) for the outermost level $|i\rangle$=$|1\rangle$,…,$|5\rangle$-, for electron configurations with up to five filled shells (Figure 1b). Blue lines: TDQMC results; green lines: numerically exact results.

Note that in the process of adding correlated electrons at the outer shells, the inner-shell electrons remain largely intact due to their stronger localization (confinement) to the core. Therefore, to a good approximation, the inner-shell wave functions need not be recalculated, and these may remain at their self-consistent Hartree–Fock configurations. Figure 4a depicts the system energy (blue line), which almost perfectly matches the numerically exact energies (green line) obtained using the standard DMC method [10]. The linear entropy predicted by the TDQMC method decreases when adding new excited states to the electron configuration (blue line in Figure 4b), which contrasts with the case of bosonic quantum dots, where it increases for more electrons at the ground state [14]. This behavior is also confirmed by the numerically exact results (green line) for levels 1 and 2 (two and four identical electrons), and can be explained by the orthogonality of the wave functions in the fermionic calculation, which causes weaker interaction for the outer-shell electrons.

## 4. Conclusions

In conclusion, we have calculated the correlated ground state and excited states of 1D quantum dots with spin-polarized and spin-compensated electron configurations with up to 5 energy levels (10 electrons) within the time-dependent quantum Monte Carlo framework. Unlike the Hartree–Fock approximation, the TDQMC method accounts for the dynamic correlations between the constituents of the quantum system, and allows one to quantify the resultant spatial entanglement in a simple and efficient manner. By variationally minimizing the system energy with respect to the spatial quantum nonlocality, the optimal set of wave functions that describes each electron is found, which allows one further to calculate the reduced density matrices for the different electrons and, hence, to quantify the entanglement that they exhibit due to their mutual interactions. Using the linear quantum entropy as a measure for the entanglement, it was found that for a fully spin-polarized electron configuration the stochastic windowing applied to the ground state alone is sufficient to recover the entanglement of the excited states, in good agreement with the exact numerical result. For the spin-compensated electron system, the entanglement that is due to the interaction of the two outermost opposite-spin electrons is dominant, while the inner shells remain largely at their Hartree–Fock states. An essential advantage of this method is that it allows one to conceive quantum particles as identical as well as distinguishable objects. The theory presented here may find useful applications in treating quantum correlation effects in composite quantum systems such as molecules, clusters, and solid-state materials. Its accuracy could be further improved by using linear combinations of Slater determinants to better approach the Hilbert space of the quantum many-body problem.

## Data Availability

Not applicable.

## References

[1] Nielsen M.A., Chuang I.L. (2010). Quantum Computation and Quantum Information.

[2] Tichy M.C., Mintert F., Buchleitner A. (2011). Essential entanglement for atomic and molecular physics. J. Phys. B Mol. Opt. Phys..

[3] Amico L., Fazio R., Osterloh A., Vedral V. (2008). Entanglement in many-body systems. Rev. Mod. Phys..

[4] Horodecki R., Horodecki P., Horodecki M., Horodecki K. (2009). Quantum entanglement. Rev. Mod. Phys..

[5] Froese-Fischer C. (1977). The Hartree-Fock Method for Atoms: A Numerical Approach.

[6] Parr R., Yang W. (1989). Density-Functional Theory of Atoms and Molecules.

[7] Alon O.E., Streltsov A.I., Cederbaum L.S. (2007). Unified view on multiconfigurational time propagation for systems consisting of identical particles. J. Chem. Phys..

[8] Szabo A., Ostlund N. (1996). Modern Quantum Chemistry.

[9] Feiguin A.E., Avella A., Mancini F. (2013). The density matrix renormalization group. Strongly Correlated Systems.

[10] Hammond B.B., Lester W., Reynolds P. (1994). Monte Carlo Methods in Ab Initio Quantum Chemistry.

[11] Christov I.P. (2006). Correlated non-perturbative electron dynamics with quantum trajectories. Opt. Express.

[12] Christov I.P. (2008). Dynamic correlations with time-dependent quantum Monte Carlo. J. Chem. Phys..

[13] Christov I.P. (2017). Particle-wave dichotomy in quantum Monte Carlo: Unlocking the quantum correlations. J. Opt. Soc. Am. B.

[14] Christov I.P. (2020). Spatial non-locality in confined quantum systems: A liaison with quantum correlations. Few Body Syst..

[15] Osenda O., Serra P. (2007). Scaling of the von Neumonn entropy in a two-electron system near the ionization threshold. Phys. Rev. A.

[16] Pont F.M., Osenda O., Toloza J.H., Serra P. (2010). Entropy, fidelity, and double orthogonality for resonance states in two-electron quantum dots. Phys. Rev. A.

[17] Nielsen E., Muller R.P., Carroll M.S. (2012). Configuration interaction calculations of the controlled phase gate in double quantum dot qubits. Phis. Rev. B.

[18] Pham D.N., Bharadwaj S., Ram-Mohan L.R. (2020). Tuning spatial entanglement in interacting two-electron quantum dots. Phys. Rev. B.

[19] Bransden B.H., Joachain C.J. (1982). Physics of Atoms and Molecules.

[20] Holland P.R. (1993). The Quantum Theory of Motion.

[21] Christov I.P. (2019). Time dependent spatial entanglement in atom-field interaction. Phys. Scr..

[22] Christov I.P. (2013). Electron-pair densities with time-dependent quantum Monte Carlo. J. Atom. Mol. Phys..

[23] Breuer H.P., Petruccione F. (2002). The Theory of Open Quantum Systems.

[24] Coe J.P., Sudbery A., D’Amico I. (2008). Entanglement and density-functional theory: Testing approximations on Hooke’s atom. Phys. Rev. B.

[25] Zanardi P., Zalka C., Faoro L. (2000). Entangling power of quantum evolutions. Phys. Rev. A.

[26] Ghirardi G., Marinatto L. (2004). General criterion for the entanglement of two indistinguishable particles. Phys. Rev. A.

[27] Plastino A.R., Manzano D., Dehesa J.S. (2009). Separability criteria and entanglement measures for pure states of N identical fermions. Europhys. Lett..

[28] Benavides-Riveros C.L., Toranzo I.V., Dehesa J.S. (2014). Entanglement in N-harmonium: Bosons and fermions. J. Phys. B At. Mol. Opt. Phys..

[29] Schliemann J., Ignacio Cirac J., Kus M., Lewenstein M., Loss D. (2001). Quantum correlations in two-fermion systems. Phys. Rev. A.

[30] Buscemi F., Bordone P., Bertoni A. (2007). Linear entropy as an entanglement measure in two-fermion systems. Phys. Rev. A.

[31] Grobe R., Eberly J.H. (1992). Photoelectron spectra for two-electron system in a strong laser field. Phys. Rev. Lett..

